# Reconfigurable, graphene-coated, chalcogenide nanowires with a sub-10-nm enantioselective sorting capability

**DOI:** 10.1038/s41378-018-0008-3

**Published:** 2018-05-21

**Authors:** Tun Cao, Long Tian, Huawei Liang, Kai-Rong Qin

**Affiliations:** 1 Department of Biomedical Engineering, Dalian University of Technology, Dalian, China; 2 Shenzhen Key Laboratory of Laser Engineering, College of Optoelectronic Engineering, Shenzhen University, Shenzhen, China

## Abstract

Chiral surface plasmon polaritons (SPPs) produced by plasmonic nanowires can be used to enhance molecular spectroscopy for biosensing applications. Nevertheless, the switchable stereoselectivity and detection of various analytes are limited by a lack of switchable, chiral SPPs. Using both finite-element method simulations and analytic calculations, we present a graphene-coated chalcogenide (GCC) nanowire that produces mid-infrared, chiral SPPs. The chiral SPPs can be reversibly switched between “on” (transparent) and “off” (opaque) by non-volatile structural state transitions in the dielectric constants of the chalcogenide glass Ge2Sb2Te5. Furthermore, by controlling the Fermi energy of the graphene-coating layer, the nanowire can output either non-chiral or chiral SPPs. A thermal-electric model was built to illustrate the possibility of ultrafast on/off switching of the SPPs at the terminus of the nanowire. Finally, we show that a selective, lateral sorting of sub-10-nm enantiomers can be achieved via the GCC nanowire. Chiral nanoparticles with opposite handedness experience transverse forces that differ in both their sign and magnitude. Our design may pave the way for plasmonic nanowire networks and tunable nanophotonic devices, which require the ultrafast switching of SPPs, and provide a possible approach for a compact, enantiopure synthesis.

## Surface plasmon polaritons: sub 10 nm enantiomer separation with nanowires

Coating of phase change Ge2Sb2Te5 nanowires with graphene enables separation of sub-10 nm enantiomers. Surface plasmon polaritons in nanowires are known to be capable of generating optical forces in different directions for the separation of chiral objects of different sign. However, modulation and switching on/off of the polaritons has been limited by the choice of nanowire. A team in China led by Tun Cao now demonstrates theoretically a graphene-coated Ge2Sb2Te5 nanowire, where the known rapid and reversible phase change – between amorphous and crystalline structures—enables a wide range of polariton modes. This contributes to the observation of sub-10 nm chiral nanoparticles of opposite handedness being pushed in different directions. This study suggests the possibility of nanowire-based enantiomer separation with enhanced nanoparticle selectivity.

## Introduction

Chiral recognition is crucial in chemical synthesis, especially in the manufacturing of pharmaceuticals^1,2^. However, the prevailing chemical methods produce redundant side products and have low discrimination^3,4^. Recently, enantiomer separation using chiral optical forces has attracted increased interest due to its advantages of being more efficient and less invasive than chemical schemes^5–9^. The idea of this optical enantiomer-selective separation is to create experimental conditions under which the chiral objects with chiral polarizabilities of opposite signs experience forces in different directions. To date, the separation of a sub-100-nm enantiomeric pair has only been numerically demonstrated using plasmonic nanostructures^10,11^, and the complicated fabrication of plasmonic nanostructures may pose limitations for further applications in the field^12–17^. Furthermore, the smallest chiral objects that have been separated thus far are much larger than the pharmaceutically relevant sub-10-nm biomolecules. It is desirable to extend this mechanism to sub-10-nm objects, e.g., chiral molecules in nature.

A recently developed metallic nanowire is a promising candidate for resolving these challenges^18–34^. The nanowire can produce chiral light and possesses a nanometer-sized geometry that is comparable to that of sub-10-nm chiral biomolecules. However, the metal component of the nanowire may result in high loss and poor biocompatibility^35^. In this regard, very recently, a graphene-coated dielectric nanowire that supports surface plasmon polariton (SPP) modes has been theoretically demonstrated^36–38^, and the SPP modes are tuned by varying the Fermi energy (*E*_F_) of the graphene coating^39–43^. The low loss and strong field confinement of the SPPs in the graphene-coated nanowire make it a better alternative than the metallic nanowire. Even so, the graphene-coated nanowires that have been investigated thus far use passive dielectric cores, such as Si and SiO_2_, which have a few disadvantages. First, little is known about actively switching the SPP waves on/off, which is an important requirement for biosensing. For example, even though the current schemes can identify the presence of chirality in molecules, the chiral expression, i.e., the magnitude of the asymmetry effects on the chemical and physical properties of the systems, is less conspicuous, which makes it more difficult to recognize, count, and modify^44–46^. Likewise, contriving biosensors capable of selectively detecting multiple analytes with different signals remains a formidable challenge. Detectors with chiroptical switches may solve this problem. Therefore, manipulating the magnitude of the chiral shape expression and related phenomena is of interest, especially if the manipulation yields an effect that can be turned “on” and “off” through external stimuli. The success of graphene-based plasmonic nanowires for manipulating SPP modes is empowered by creative designs and advanced nano-fabrications^36–43^, but very few of these studies have investigated the modulation rate of the SPP modes. It is crucial to recognize that these limitations can be avoided if the core of the nanowire has an ultrafast reconfigurability. Chalcogenide glass exhibits a remarkable portfolio of properties^47^, and Ge_2_Sb_2_Te_5_ (GST) is important due to its fast switching speed, excellent thermal stability, and high cyclability. In particular, the rapid and reversible phase transition of GST between amorphous and crystalline^48,49^ makes the material ideal for rapidly tunable photonic devices^50–52^. The GST nanowire has proved to be promising and useful for high-density memory devices since it has a low-power consumption and writing currents^53–58^. Our research extends the above knowledge into the field of chiral recognition, i.e., enantioselective optical separation.

In this work, we present lateral sorting of paired sub-10-nm enantiomers by combining chiral transverse forces with a graphene-coated GST (GCG) nanowire. First, we designed a GCG nanowire to produce chiral SPPs by introducing a coherent superposition of transverse magnetic (TM) and electromagnetic hybrid (EH) SPPs with right-/left-handed quasi-elliptically polarized (QEP) states at the input. By mismatching the mode-phase-matching (MPM) condition, the coherent superposition of the different SPP modes causes the light to propagate along a helical path, producing an unstable helix crescent light (chiral SPP). We show that the reversible phase transition between the amorphous GST and crystal provides a significant contrast in the real part of the permittivity and allows switching of the output chiral SPPs. The SPP modes can be interchanged between the chiral and non-chiral beam profiles via the Fermi energy (*E*_F_) of the graphene coating. Herein, *E*_F_ varies in an appropriate region from 0.46 to 0.6 eV. The chiral SPP mode operates across a broad spectral range of 3 to 5 μm. The GST phase change material possesses a crystallization temperature of *T*_C_ = 433 K and a melting temperature of *T*_M_ = 873 K. The as-deposited amorphous GST can be crystallized by heating it beyond the *T*_C_ instead of below the *T*_M_. A reverse re-amorphization process (from the crystalline to the amorphous state) can be obtained by rapidly increasing the local temperature above *T*_M_^59^. Our thermal-electric model shows that the temperature of the GST rod increased from room temperature to 433 K in 2.4 ns (8 ns) with a biasing voltage of *V*_g_ = 17 V (10 V), which can switch off the chiral (non-chiral) SPPs. The SPPs can be switched on again in 5 ns during the re-amorphization with *V*_g_ = 25 V. When the state of the GST core is amorphous and the MPM state is mismatched by *E*_F_ = 0.6 eV, the sub-10-nm paired enantiomers placed 5 nm above the output plane of the nanowire experience an opposite, large chiral transverse force (~300 fN) under a low incident light intensity (~100 mW/μm^2^). This allows the chiral force to push chiral nanoparticles with opposite handedness in opposite directions. The emissive chiral light can be terminated by changing the GST from amorphous to crystalline, which disables the enantiomer separation.

## Results

**Fig. 1:**
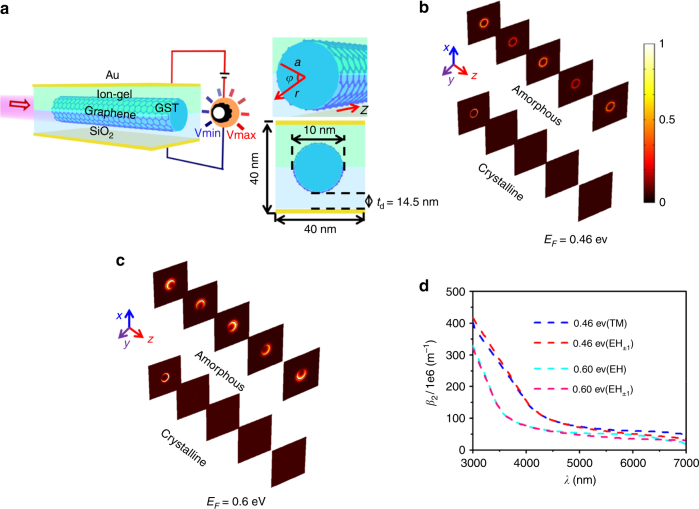
**a** Sketch of the GCG nanowire. The nanowire described in a cylindrical coordinate system is shown in the top inset and its cross-section is shown in the bottom one. Switching “on” (top panel) and “off” (bottom panel) **b** non-chiral SPP modes at *E*_F_ = 0.46 eV and **c** chiral SPP modes at *E*_F_ = 0.6 eV by changing the GST phase from amorphous to crystalline. Fig. 1b,c share the same color bar. **d** Comparison of *β*_2_ for the EH_±1_ and TM SPP modes in the GCG nanowire with the crystalline GST core

Figure 1a shows a sketch of a GCG nanowire with a radius of 5 nm (*a* = 5 nm) and a 0.5-nm-thick graphene coating (*T*_g_ = 0.5 nm). *V*_g_ was applied to the graphene coating via an ion gel with an index that matches that of the SiO_2_ (*ε*_gel_ = 2.25) layer. The *E*_F_ was changed through a field effect transistor (FET)^60^, and a change in *V*_g_ of a few volts leads to a variation of 0.1 eV in *E*_F_. The change in *E*_F_ can modulate the graphene permittivity, *ε*_0_. This controls the beam profile of the SPPs that propagate through the GCG nanowire. The graphene is defined as an atom-thick anisotropic sheet with $\varepsilon _{\rm g} = 1 + \frac{{i\sigma _{\rm g}}}{{\omega \varepsilon _0T_{\rm g}}}$, where *σ*_g_ represents the surface conductivity of graphene and *ε*_0_ the air permittivity^61^. The Kubo formula is used to express *σ*_g_^62^. σ_g_ = σ_intra_ + σ_inter_ is composed of the intraband conductivity, $\sigma _{{\mathrm{intra}}} = \frac{{ie^2k_{\mathrm{B}}T}}{{\pi h^2(\omega + i2\Gamma )}}\{ \frac{{E_F}}{{k_{\mathrm{B}}T}} + 2\ln [\exp ( - \frac{{E_F}}{{k_{\mathrm{B}}T}}) + 1]\}$, and the interband conductivity, $\sigma _{{\mathrm{inter}}} = {\int}_0^\infty {\frac{{ie^2(\omega + i2\Gamma )}}{{\pi h^2}}} \frac{{[\exp (\frac{{ - \varepsilon - E_{\rm {F}}}}{{k_{\mathrm{B}}T}}) + 1]^{ - 1} - [\exp (\frac{{\varepsilon - E_{\rm {F}}}}{{k_{\mathrm{B}}T}}) + 1]^{ - 1}}}{{(\omega + i2\Gamma )^2 - 4(\varepsilon /h)^2}}d\varepsilon$, where the phenomenological scattering rate is *Γ* = 0.1 meV, *T* is the temperature, *e* is the electron charge, *ħ* is the reduced Planck’s constant, and *k*_B_ is the Boltzmann constant, and *f*_d_(*ε*) = $1+{\mathrm {e}}^{({\epsilon}-E_{\mathrm {F}})/(k_{\mathrm{B}}T) -1}$ is the Fermi–Dirac distribution. *E*_F_ is determined by the carrier density of graphene, $n_{\rm s} = \frac{1}{{\pi h^2v_{\rm F}^2}}\mathop {\int}\limits_0^\infty {[f_{\rm d}\left( \varepsilon \right) - f_{\rm d}\left( {\varepsilon + 2E_{\rm F}} \right)]} \varepsilon d\varepsilon$, where *v*_F_ = 9.5 × 10^5^ m/s is the Fermi velocity. *n*_s_ can also be expressed as *n*_s_ = *V*_g_*ε*_gel_*ε*_0_/*et*_d_, where *t*_d_ = 14.5 nm and *ε*_gel_ = 2.25 are the thickness and permittivity of the ion–gel layer, respectively^63^. Derived from the two different expressions of *n*_s_, the value of *V*_g_ in the dependence on *E*_F_ can be obtained as $V_{\rm g} = \frac{{et_{\rm d}\left( {eE_{\rm F}} \right)^2}}{{2\varepsilon _0\varepsilon _{\rm {gel}}\pi h^2v_{\rm F}^2}} + E_{\rm F}$. Figure S1 in the Supporting Information (SI) shows the complex dielectric constants of GST for both the amorphous (red line) and crystalline (blue line) states that were experimentally measured^49^. The radical change in the dielectric constant between the structural states is observed over a wide mid-infrared (M-IR) region; for example, *ε*_a-GST_ ≈ 16 + 0.05_*i*_, *ε*_c-GST_ ≈ 35 + 0.2_*i*_ from 3 to 7 μm. *ε*_a-GST_ and *ε*_c-GST_ represent the GST permittivities for the amorphous and crystalline states, respectively.

The GCG nanowire can simultaneously produce and propagate the QEP EH, quasi-linearly polarized (QLP) EH, and TM plasmon modes, where the distributions of the time-averaged Poynting vectors of the plasmon modes, $\left\langle S \right\rangle = {\Re} \left[ {E_rH_\varphi ^ \ast - E_\varphi H_r^ \ast } \right]/2$, are numerically demonstrated in Fig. S2 of the SI^64^. It is noteworthy that these plasmon modes can be experimentally produced by hybrid plasmonic waveguides^65^ and tapered graphene^66^. The analysis of the electromagnetic (EM)-field components for both the EH and TM SPPs is shown in S3 of the SI. The polarization properties of QEP EH_−1_ and QEP EH_+1_ and the QLP plasmon modes are analytically investigated in S4, where QEP EH_+1_ and QEP EH_−1_ represent the <S> of the right and left-handed QEP EH modes, respectively. As seen in Fig. S2 and S4, the polarization behaviors of the EH and TM plasmon modes propagating through the GCG nanowire are very different. Their coherent superposition leads to either constructive or destructive interference, which is related to the variation in the propagation constant. The propagation constant, *β* = *β*_1_ + *β*_2_*i*, of the SPP modes is strongly dependent on the interplay between the GST phase transition and the change in *E*_F_ in the GCG nanowire. In the top panel of Fig. 1b, we show that the GCG can propagate a stable, non-chiral beam launched by the coherent superposition of the incident TM and QEP EH modes, where the state of the GST core is amorphous and *V*_g_ = 10 V, corresponding to *E*_F_ = 0.46 eV. Herein, the TM and QEP EH modes possess identical values of *β*_1_. This causes the operation wavelength, *λ*_oper_, to reach the MPM condition (*λ*_oper_ = *λ*_MPM_ = 4 μm). As a result, the coherent interference of the two modes can support a ring beam propagating along the GCG nanowire (see Fig. S5 and S3 of SI). A phase change of the GST from amorphous to crystalline can switch off the output ring beam (the bottom panel of Fig. 1b). In the top panel of Fig. 1c, we increase *V*_g_ to 17 V (*E*_F_ = 0.6 eV), which is applied to the GCG nanowire with an amorphous GST core. This illustrates that the GCG nanowire can export a helix crescent light. This is because *λ*_oper_ = 4 μm is not located at the MPM point (*λ*_MPM_ = 3.5 μm) at *E*_F_ = 0.6 eV, and the beam can be transported along a helical path (see S5 and Fig. S3 of the SI). Likewise, the output chiral beam with a helix crescent profile can be switched off by changing the GST state to crystalline (the bottom panel of Fig. 1c). Such a GCG nanowire acts as a reconfigurable SPP mode converter for the non-chiral and chiral states, which is mainly achieved by tuning the *E*_F_ (*V*_g_) of graphene, and exhibits a switchable function via the GST phase transition. In Fig. S3 of the SI, we calculate and discuss the complex propagation constants, *β* = *β*_1_ + *β*_2_*i*, of the TM and EH_±1_ SPPs propagating along the GCG nanowire at *E*_F_ = 0.46 and 0.6 eV when the GST core is amorphous. Moreover, Fig. 1d further explains how the crystallization of the GST core switches off the output SPP modes. As seen, the values of *β*_2_ for the SPP modes propagating along the crystalline GCG nanowire are approximately two orders of magnitude greater than those of the amorphous one (Fig. S3(a) of the SI). This is particularly attractive because the larger *β*_2_ can considerably attenuate the SPP modes. Thus, by switching the state of GST between amorphous and crystalline, the SPPs can be either “on” (transparent) or “off” (opaque). This further explains the observation from Fig. 1b,c. A simulation was carried out using the commercial finite-element method package COMSOL. A detailed description of the model can be found in S6 of the SI. As an *E*_F_ beyond 0.7 eV is difficult to achieve in a real experiment^67^, we controlled *E*_F_ over a realistic range of 0.46 to 0.6 eV.

**Fig. 2:**
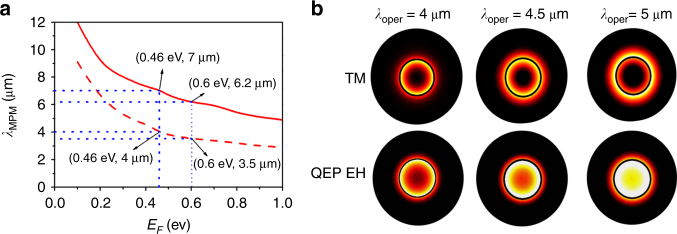
**a** Spectra of *λ*_MPM_ versus *E*_F_ for the GCG nanowire with amorphous GST, and the dashed and solid red lines represent the shorter and longer *λ*_MPM_, respectively. **b** The <S> of the TM modes (top panel) and QEP EH modes (bottom panel) at the output of the GCG nanowire for *λ*_oper_ = 4 μm, *λ*_oper_ = 4.5 μm, and *λ*_oper_ = 5 μm

In Fig. 2a, we investigated the dependence of the MPM wavelengths on the *E*_F_ for the GCG nanowire with amorphous GST, and the solid and dashed lines show the longer and shorter MPM wavelengths, respectively. At each given *E*_F_, we calculated the wavelength-dependent *β*_1_ spectra for both the TM and EH_±1_ modes (left column of Fig. S3(a) of the SI). Two MPM wavelengths are observed when the *β*_1_ values of the different modes are identical (right column of Fig. S3(a) of the SI). For example, the MPM wavelengths are *λ*_MPM_ = 4 and 7 μm at *E*_F_ = 0.46 eV, but only the shorter wavelength of *λ*_MPM_ = 4 μm can be used. To further explore this concept, in Fig. 2b the values of <S> of the TM and QEP EH modes were examined at several wavelengths. As seen in the top panel of Fig. 2b, the <S> of the TM mode is approximately zero inside the GST core over a broad spectral region. However, the <S> of the QEP EH mode in the center increases with *λ*_oper_ (bottom panel of Fig. 2b). Therefore, field overlapping, which would produce a stable ring beam with *λ*_oper_ > 4.5 μm, is not achieved. Namely, the overlap of the TM and QEP EH modes can be efficiently preserved with *λ*_oper_ < 4.5 μm. This is because $\beta _2^{{\rm {EH}}_{ \pm 1}}$coincides with *β*_2_^TM^ (namely, small$\Delta \beta _2 = \beta _2^{{\rm {EH}}_{ \pm 1}} - \beta _2^{\rm {TM}}$) across a spectral region from 3 to 4.5 μm (right column of Fig. S3(b) of SI), which, in turn, provides strong mode interference.

In Fig. S4 of the SI, we calculated the degree of circular polarization, $C = \frac{{2\left\langle {E_x\left( t \right)E_z\left( t \right)\sin \left( {\delta _x - \delta _z} \right)} \right\rangle }}{{\left\langle {E_x^2\left( t \right) + E_y^2\left( t \right) + E_z^2\left( t \right)} \right\rangle }}$^1^, where *δ*_*x*_ – *δ*_*z*_ is the phase difference between the transverse *E*-field components *E*_*x*_ and *E*_*z*_ and <> denotes the time average. The calculation shows that the photons emitted by the GCG nanowire possess a relatively high *C* (*C* > 0.5) over most of the M-IR regime.

**Fig. 3:**
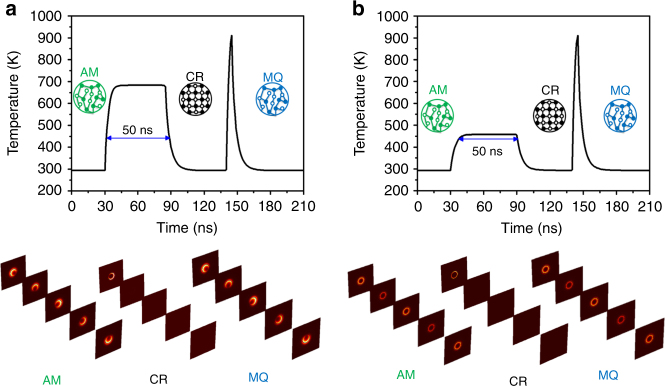
**a** Switching the chiral SPP modes. The as-deposited amorphous (AM) GST core is electrically heated beyond *T*_C_ = 433 K, allowing it to transform into crystalline (CR) GST under *V*_g_ = 17 V. *V*_g_ = 25 V is applied to heat the CR-GST beyond *T*_M_ = 873 K. Subsequent quenching leads to the melt-quenched (MQ) state GST. **b** Switching of the non-chiral SPP modes, where the AM-GST rod is electrically heated above *T*_C_ = 433 K by using *V*_g_ = 10 V

The structural state of GST changes from amorphous to crystalline (crystallization) upon heating to a temperature between the *T*_C_ and *T*_M_. This phase transition is reversible (re-amorphization) if the local temperature is momentarily increased above the *T*_M_. Recently, it has been shown that the phase change speed of GST is faster than 100 ns^68^. In Fig. 3a,b, a heat transfer model was built to explore the temporal change in the temperature of the GST core at *V*_g_ = 17 and 10 V, respectively. In Table S1 of the SI, we summarize the material thermoelectric properties that were used to define the graphene, ion gel, and GST in the thermal-electric model. The temperature of the as-deposited amorphous GST rod rapidly increases with the time of loading *V*_g_ = 17 V (Fig. 3a), and the chiral SPP modes are excited due to *E*_F_ = 0.6 eV. The temperature increases above *T*_C_ = 433 K after 2.4 ns, crystallizing the GST. A subsequent annealing procedure is performed to maintain the temperature above *T*_C_ but below *T*_M_ for ~50 ns^69^. This can fully crystallize the GST and completely turn off the chiral SPP modes. The temperature of the crystalline GST core decreases to room temperature once *V*_g_ = 17 V is removed due to heat dissipation into the air. The re-amorphization of the GST can turn on the chiral SPP modes. To re-amorphize the GST, the crystal lattice needs to be molten and subsequently quenched to room temperature to avoid recrystallization of the atomic structure. A biasing time of 5 ns at *V*_g_ = 25 V was selected to re-amorphize the GST. *V*_g_ = 25 V can provide a high thermal energy that rapidly increases the temperature above the *T*_M_, melting the GST. By switching off *V*_g_ = 25 V, the subsequent fast cooling can quench the melt in an amorphous state. The chiral SPP mode was switched on again by setting *V*_g_ back to 17 V. Figure 3b shows the temporal variations in the GST temperature under *V*_g_ = 10 V (*E*_F_ = 0.46 eV), and the reversible switching of non-chiral SPP modes was obtained. Moreover, videos 1 and 2 record the whole process of turning “on/off” the chiral and non-chiral SPP modes, respectively, which showed that our GCG nanowire exhibits an excellent performance for dynamically switchable functions.

A nanometer-sized chiral specimen interfering with a monochromatic EM field can be characterized by oscillating magnetic and electric dipolar moments, **M** = Re[**m**(*r*)*e*^−*iωt*^] and *P* = Re[**p**(**r**)**e**^−*iωt*^], respectively. The dipolar moments **m** and **p** are proportional to the local magnetic, **H**, and electric, **E**, fields at the target object, which are expressed as:^70,71^

1 $$\left( {\begin{array}{*{20}{c}} p \\ m \end{array}} \right) = \left( {\begin{array}{*{20}{c}} {\alpha \varepsilon _{\rm {d}}} & {{\it{i}}\chi \sqrt {\varepsilon _{\rm {d}}\mu _{\rm {d}}} } \\ { - {\it{i}}\chi \sqrt {\varepsilon _{\rm {d}}\mu _{\rm {d}}} } & {\beta \mu _{\rm {d}}} \end{array}} \right) \times \left( {\begin{array}{*{20}{c}} E \\ H \end{array}} \right)$$

where *μ*_d_ and *ε*_d_represent the permeability and permittivity of the surrounding medium, respectively. The magnetic, *β*, electric, *α*, and mixed magnetic-electric, *χ*, dipolar polarizabilities are complex scalars, where the sign (+,−) of *χ* is associated with the handedness of the enantiomers. It is noteworthy that *β* and *α* are the same for two enantiomers with opposite handedness, since they are quadratic forms of the magnetic and electric dipoles^72^. Therefore, our task is to distinguish the effect of the sign change of *χ* on the optical forces exerted on the chiral object. For a nanosphere, *α*, *β*, and *χ* can be represented as:^73^

2 $$\alpha = 4\pi {\it{R}}^3\frac{{\left( {\varepsilon _{\rm {r}} - 1} \right)\left( {\mu _{\rm {r}} + 2} \right) - \kappa ^2}}{{\left( {\varepsilon _{\rm {r}} + 2} \right)\left( {\mu _{\rm {r}} + 2} \right) - \kappa ^2}}$$

3 $$\beta = 4\pi {\it{R}}^3\frac{{\left( {\mu _{\rm {r}} - 1} \right)\left( {\varepsilon _{\rm {r}} + 2} \right) - \kappa ^2}}{{\left( {\varepsilon _{\rm {r}} + 2} \right)\left( {\mu _{\rm {r}} + 2} \right) - \kappa ^2}}$$

4 $$\chi = 12\pi {\it{R}}^3\frac{\kappa }{{\left( {\varepsilon _{\rm {r}} + 2} \right)\left( {\mu _{\rm {r}} + 2} \right) - \kappa ^2}}$$

*μ*_r_ = *μ*/*μ*_d_ and *ε*_r_ = *ε/ε*_d_ are the relative permeability and permittivity relating to the surrounding media, respectively; (*ε*, *μ*) provides the object’s refractive index, *n*; *κ* is the object’s chirality, and *R* is the object’s radius.

The time-averaged chiral force, F_*χ*_, acting on a chiral specimen is composed of reactive ($F_\chi ^{\rm {reac}}$) and dissipative ($F_\chi ^{\rm {diss}}$) constituents, which are associated with the real and imaginary parts of the complex, *χ*, respectively:^70,74^

5 $${\bf{F}}_\chi = {\Re} \left[ \chi \right] \cdot \frac{{\it{c}}}{\omega }\nabla K + {\Im} \left[ \chi \right] \cdot \frac{2}{{\it{c}}}\left( {\Phi - \frac{{\nabla \times {\Pi}}}{2}} \right)$$

where $F_\chi ^{\rm {reac}} = {\Re} \left[ \chi \right] \cdot \frac{{\it{c}}}{\omega }\nabla K$, $F_\chi ^{\rm {diss}} = {\Im} \left[ \chi \right] \cdot \frac{2}{{\it{c}}}\left( {\Phi - \frac{{\nabla \times {\Pi}}}{2}} \right)$, ${\Pi} = \frac{{{\Re} \left[ {E \times H^ \ast } \right]}}{2}$is the Poynting vector, ${K} = \frac{{{\Im} \left[ {{\bf{E}} \cdot {\bf{H}}^ \ast } \right]\omega }}{{2c^2}}$ is the chirality density of the EM-field, and *ω* is the angular frequency of the EM wave.

**Fig. 4:**
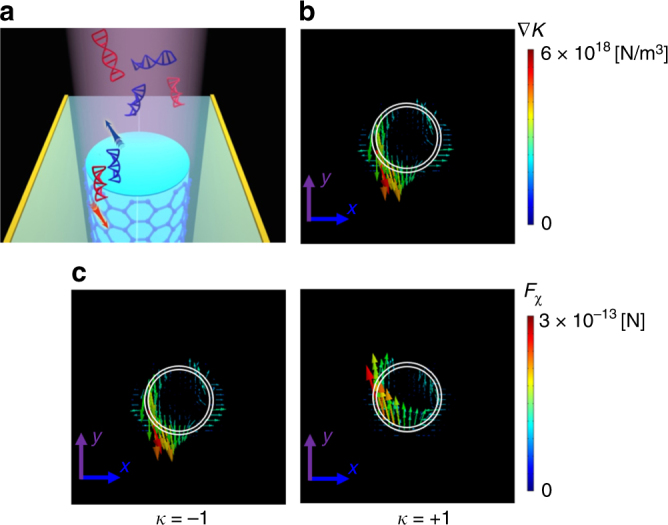
**a** Illustration of the GCG nanowire, where the enantiomeric pair is placed 5 nm above the output plane of the nanowire. The GCG nanowire is launched by a coherent superposition of the TM SPP and EH SPP modes at the input with an incident light intensity of 100 mW/μm^2^. **b** The distribution of ∇*K* at *λ*_oper_ = 4 μm. **c** **F***χ* exerted on the chiral nanoparticles (*R* = 3 nm) with *κ* = −1 (left column) and *κ* = + 1 (right column) at *λ*_oper_ = 4 μm. The white solid lines are used to outline the structure’s geometry. The direction and color of the arrows represent the direction and magnitude of ∇*K* and **F***χ*, respectively

The flow of the chirality is expressed by$\Phi = \frac{{\omega \left( {\varepsilon _{\rm {d}}\Phi _{\rm {E}} + \mu _{\rm {d}}\Phi _{\rm {H}}} \right)}}{2}$, where $\Phi _{\rm {E}} = - \frac{1}{2}{\Im} \left[ {E \times E^ \ast } \right]$ and $\Phi _{\rm {H}} = - \frac{1}{2}{\Im} \left[ {H \times H^ \ast } \right]$. Herein, to simplify the calculation, we did not take into account the imaginary part, *κ*, of the chiral specimens by assuming that Im(*κ*) ≪ Re(*κ*)^8,11^. Therefore, $F_\chi ^{\rm {diss}}$can be ignored due to the absence of the Im[*χ*]of the chiral entity, and F_*χ*_ is mainly determined by the gradient of the optical chirality (∇*K*). Figure 4a schematically illustrates an enantiomer-selective separation of the chiral specimens with opposite handedness using the GCG nanowire, where *λ*_oper_ = 4 μm, *E*_F_ = 0.6 eV, and the state of the GST core is amorphous. The radius of a chiral nanoparticle is *R* = 3 nm. The chirality is *κ* = ± 1, where ‘−’ and ‘+’ denote the left-handed and right-handed enantiomers, respectively. The paired enantiomers are located 5 nm above the output plane of the nanowire. The chiral molecules are in the air. The input light intensity of the nanowire is 100 mW/μm^2^. Upon illumination with the coherent superposition of the EH and TM SPP modes, we show the ∇*K* as arrows in Fig. 4b, and the direction and color of the arrows represent the direction and strength of ∇*K*, respectively. Figure 4c presents F_*χ*_ acting on the enantiomeric pair in the *x–y* plane. The left column of Fig. 4c shows that for a chiral specimen with *κ* = −1, F_*χ*_ mainly drags the specimen downwards. For a specimen with *κ* = + 1, F_*χ*_ mostly repels the particles upwards (right column of Fig. 4c). This is very close to the ideal experimental circumstance; i.e., sub-10-nm chiral molecules (*R* = 3 nm) with opposite handedness above the output plane of the nanowire can experience opposite and relatively large F_*χ*_ (~300 fN) under a low incident light intensity (~100 mW/μm^2^). That, in turn, pushes the chiral molecules with opposite handedness in different directions. However, the enantioselective separation of the chiral specimens is deactivated by crystallizing the GCG nanowire.

**Fig. 5:**
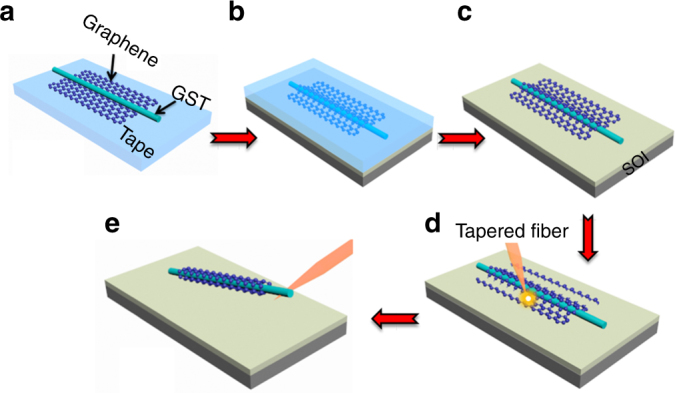
Schematic illustration of the GCG nanowire fabrication process. **a** A GST nanowire was placed above a graphene sheet on scotch tape. **b** The tape with the graphene and GST nanowire was attached to an SOI substrate. **c** By removing the tape in a 4-methyl-2-pentanone solution, a GCG nanowire on a SOI substrate was obtained. **d** The graphene sheet was cut along the nanowire by a nanosecond pulse laser propagating through the tapered fiber. **e** The GCG nanowire was removed from the wafer by the tapered fiber

Figure 5a–e schematically illustrate the fabrication process of the GCG nanowire. A graphene sheet was pulled away from Kish graphite using adhesive tape and adhered to the tape^41^. The GST nanowire was synthesized by a catalyst-mediated vapor–liquid–solid process and was placed on the graphene sheet using micromanipulation (Fig. 5a)^54^. Afterwards, the tape was attached to a silicon-on-insulator (SOI) wafer, and the side with the nanowire faced the wafer (Fig. 5b). We immersed the wafer in a 4-methyl-2-pentanone solution to dissolve the tape. Thus, a GCG nanowire on a SOI substrate was obtained (Fig. 5c). Then, a nanosecond laser beam propagating through a nanoscale tapered fiber was used to cut the graphene sheet along both sides of the nanowire on the substrate (Fig. 5d). Finally, the nanowire was removed from the substrate using the tapered fiber, and graphene spontaneously enfolded the nanowire to create a GCG nanowire (Fig. 5e).

## Conclusions

This work has expanded the possibility of using GCG nanowire design to develop an optical separation process for sub-10-nm paired enantiomers. By introducing a nanowire with a coherent superposition of the TM and QEP EH modes, our proposed GCG nanowire can produce both an unstable helix crescent beam (chiral SPP) with *E*_F_ = 0.6 eV and a stable ring beam (non-chiral SPP) with *E*_F_ = 0.46 eV. In particular, the output chiral SPP modes will exert opposite chiral forces on the enantiomers at the boundary of the nanowire in opposite directions to obtain the enantioseparation. Our thermal model shows that this nanowire is dynamically reconfigurable, and the output SPP modes can be switched on/off by changing the phase of the GST core between amorphous and crystalline. Our findings suggest that the GCG nanowire can be used with a chiral source to separate sub-10-nm chiral molecules and integrated with a subwavelength photonic device to allow ultrafast optical switching, which may be a critical application for plasmonic nanowire networks.

## Electronic supplementary material

Reconfigurable Graphene-Coated Chalcogenide Nanowire with Sub-10-nm Enantioselective Sorting Capability(DOCX 934 kb)

video1

video2
