# Supplementary material for: Reconfigurable, graphene-coated, chalcogenide nanowires with a sub-10-nm enantioselective sorting capability
Source: Microsyst Nanoeng. 2018 May 21;4:7. doi: 10.1038/s41378-018-0008-3 (PMC6220155; doi:10.1038/s41378-018-0008-3)
Supplement: Supplementary file 1 — Reconfigurable Graphene-Coated Chalcogenide Nanowire with Sub-10-nm Enantioselective Sorting Capability(DOCX 934 kb) [file 41378_2018_8_MOESM1_ESM.docx]

Supporting Information

Reconfigurable Graphene-Coated Chalcogenide Nanowire with Sub-10-nm Enantioselective Sorting Capability

Tun Cao*, Long Tian, Huawei Liang, and Kai-Rong Qin

^*^E-mail: [caotun1806@dlut.edu.cn](mailto:caotun1806@dlut.edu.cn)

**S1. The dielectric constants of Ge_2_Sb_2_Te_5_ (GST) at the different structural states**

Fig. S1. Complex dielectric constants of GST, where the $\varepsilon_{a-GST}$ and $\varepsilon_{c-GST}$ represent the permittivity of the GST for both amorphous and crystalline states, respectively.

**S2. The time-averaged Poynting vectors <S> of the guided plasmon modes in the graphene-coated Ge_2_Sb_2_Te_5_ (GCG) nanowire**

The <S> of QEP EH_+1_ and QEP EH_−1_ possess the same profile of ring beam, where “+” and “-” represent the right- and left- handed QEP EH modes, respectively. The beam profile of QEP EH modes is also similar to the TM mode. The coherent overlap of QEP EH_−1_ and QEP EH_+1_ modes with the identical magnitude and phase provide a QLP EH mode. Conversely, a QEP EH mode is constructed by a coherent superposition of the two orthotropic QLP EH modes.

Fig. S2. The <S> of the guided plasmon modes in the GCG nanowire for the (a) TM, (b) QLP EH, and (c) QEP EH modes.

**S3. Electromagnetic (EM) field expressions of EH and TM modes**

**S3.1 EH Modes**

**S3.1.1 Inside the nanowire ($r<a$)**

$E_{z}^{in}=A_{E}^{in}I_{m}(h_{in}r)\Phi$ (s1)

$E_{r}^{in}=A_{E}^{in}\frac{-i\beta}{h_{in}^{2}}[h_{in}I'_{m}(h_{in}r)+i\frac{\omega\mu_{0}\mu_{a-GST}m^{2}}{ra}\frac{\frac{ih_{out}}{\omega}(\frac{1}{h_{in}^{2}}-\frac{1}{h_{out}^{2}})\frac{I_{m}(h_{in}a)}{K'_{m}(h_{out}a)}-\frac{\sigma_{\text{g}}}{h_{in}^{2}}\frac{I_{m}(h_{in}a)}{K_{m}(h_{out}a)}}{\frac{i\sigma_{\text{g}}\omega}{h_{in}}\frac{I'_{m}(h_{in}a)}{K_{m}(h_{out}a)}-\frac{I_{m}(h_{in}a)}{K_{m}(h_{out}a)}+\frac{h_{out}}{h_{in}}\frac{I'_{m}(h_{in}a)}{K'_{m}(h_{out}a)}}I_{m}(h_{in}r)]\Phi$ (s2)

(s3)

$H_{z}^{in}=A_{E}^{in}\frac{\beta m}{a}\frac{\frac{ih_{out}}{\omega}(\frac{1}{h_{in}^{2}}-\frac{1}{h_{out}^{2}})\frac{I_{m}(h_{in}a)}{K'_{m}(h_{out}a)}-\frac{\sigma_{\text{g}}}{h_{in}^{2}}\frac{I_{m}(h_{in}a)}{K_{m}(h_{out}a)}}{\frac{i\sigma_{\text{g}}\omega}{h_{in}}\frac{I'_{m}(h_{in}a)}{K_{m}(h_{out}a)}-\frac{I_{m}(h_{in}a)}{K_{m}(h_{out}a)}+\frac{h_{out}}{h_{in}}\frac{I'_{m}(h_{in}a)}{K'_{m}(h_{out}a)}}I_{m}(h_{in}r)\Phi$ (s4)

$H_{r}^{in}=A_{E}^{in}\frac{-m}{h_{in}^{2}}[\frac{\omega\varepsilon_{0}\varepsilon_{a-GST}}{r}I_{m}(h_{in}r)+\frac{i\beta^{2}h_{in}}{a}\frac{\frac{ih_{out}}{\omega}(\frac{1}{h_{in}^{2}}-\frac{1}{h_{out}^{2}})\frac{I_{m}(h_{in}a)}{K'_{m}(h_{out}a)}-\frac{\sigma_{\text{g}}}{h_{in}^{2}}\frac{I_{m}(h_{in}a)}{K_{m}(h_{out}a)}}{\frac{i\sigma_{\text{g}}\omega}{h_{in}}\frac{I'_{m}(h_{in}a)}{K_{m}(h_{out}a)}-\frac{I_{m}(h_{in}a)}{K_{m}(h_{out}a)}+\frac{h_{out}}{h_{in}}\frac{I'_{m}(h_{in}a)}{K'_{m}(h_{out}a)}}I'_{m}(h_{in}r)]\Phi$ (s5)

(s6)

where .

**S 3.1.2 Outside the nanowire ($r>a$)**

$E_{z}^{out}=A_{E}^{in}\frac{I_{m}(h_{in}a)}{K_{m}(h_{out}a)}K_{m}(h_{out}r)\Phi$ (s7)

$E_{r}^{out}=A_{E}^{in}\frac{i\beta}{h_{out}^{2}}[-\frac{I_{m}(h_{in}a)}{K_{m}(h_{out}a)}h_{out}K'_{m}(h_{out}r)+i\frac{\omega\mu_{0}\mu_{SiO_{2}}m^{2}}{ra}\frac{\frac{ih_{in}}{\omega}(\frac{1}{h_{in}^{2}}-\frac{1}{h_{out}^{2}})\frac{I_{m}(h_{in}a)}{I'_{m}(h_{in}a)}+\frac{\sigma_{\text{g}}}{h_{in}^{2}}\frac{I_{m}(h_{in}a)}{\frac{i\sigma_{\text{g}}\omega}{h_{in}}I'_{m}(h_{in}a)-I_{m}(h_{in}a)}}{\frac{K_{m}(h_{out}a)}{\frac{i\sigma_{\text{g}}\omega}{h_{in}}I'_{m}(h_{in}a)-I_{m}(h_{in}a)}+\frac{h_{in}}{h_{out}}\frac{K'_{m}(h_{out}a)}{I'_{m}(h_{in}a)}}K_{m}(h_{out}r)]\Phi$ (s8) (s9)

$H_{z}^{out}=A_{E}^{in}\frac{-\beta m}{a}\frac{\frac{ih_{in}}{\omega}(\frac{1}{h_{in}^{2}}-\frac{1}{h_{out}^{2}})\frac{I_{m}(h_{in}a)}{I'_{m}(h_{in}a)}+\frac{\sigma_{\text{g}}}{h_{in}^{2}}\frac{I_{m}(h_{in}a)}{\frac{i\sigma_{\text{g}}\omega}{h_{in}}I'_{m}(h_{in}a)-I_{m}(h_{in}a)}}{\frac{K_{m}(h_{out}a)}{\frac{i\sigma_{\text{g}}\omega}{h_{in}}I'_{m}(h_{in}a)-I_{m}(h_{in}a)}+\frac{h_{in}}{h_{out}}\frac{K'_{m}(h_{out}a)}{I'_{m}(h_{in}a)}}K_{m}(h_{out}r)\Phi$ (s10)

$H_{r}^{out}=A_{E}^{in}\frac{im}{h_{out}^{2}}[\frac{i\omega\varepsilon_{0}\varepsilon_{SiO_{2}}}{r}\frac{I_{m}(h_{in}a)}{K_{m}(h_{out}a)}K_{m}(h_{out}r)+\frac{\beta^{2}h_{out}}{a}\frac{\frac{ih_{in}}{\omega}(\frac{1}{h_{in}^{2}}-\frac{1}{h_{out}^{2}})\frac{I_{m}(h_{in}a)}{I'_{m}(h_{in}a)}+\frac{\sigma_{\text{g}}}{h_{in}^{2}}\frac{I_{m}(h_{in}a)}{\frac{i\sigma_{\text{g}}\omega}{h_{in}}I'_{m}(h_{in}a)-I_{m}(h_{in}a)}}{\frac{K_{m}(h_{out}a)}{\frac{i\sigma_{\text{g}}\omega}{h_{in}}I'_{m}(h_{in}a)-I_{m}(h_{in}a)}+\frac{h_{in}}{h_{out}}\frac{K'_{m}(h_{out}a)}{I'_{m}(h_{in}a)}}K'_{m}(h_{out}r)]\Phi$ (s11)

(s12)

**S 3.2 TM SPPs**

**S 3.2.1 Inside the nanowire ($r<a$)**

(s13)

$E_{r}^{in}=-\frac{i}{h_{in}}A_{E}^{in}\beta I_{1}(h_{in}r)\Phi_{0}$ (s14)

(s15)

where $\Phi_{0}=e^{-i(\omega t-\beta z)}$.

**S 3.2.2 Outside the nanowire ($r>a$)**

$E_{z}^{out}=A_{E}^{in}\frac{I_{0}(h_{in}a)}{K_{0}(h_{out}a)}K_{0}(h_{out}r)\Phi_{0}$ (s16)

$E_{r}^{out}=A_{E}^{in}\frac{i\beta}{h_{out}}\frac{I_{0}(h_{in}a)}{K_{0}(h_{out}a)}K_{1}(h_{out}r)\Phi_{0}$ (s17)

(s18)

**S 4. Polarization properties of EH SPPs**

**S 4.1 EH_+1_ SPPs**

**S 4.1.1 Inside the nanowire**

*ϕ* = 0^0^ represents the horizontal right direction. Based on the numerical calculation of the *E*-field distributions, the phase of the azimuthal *E*-field (denoted as) is π/2, larger than that of the radial *E*-field (denoted as$E_{r}^{in}$). Therefore, the horizontal and perpendicular components of *E*-fields with the different directions *ϕ* at *z* = 0, is expressed as

(s19)

and

(s20)

The intersection angle *θ_in_* between the polarization directions of the horizontal and perpendicular *E*-field satisfies the equation:

(s21)

At , Eq. (s21) can be further simplified as

$\tan\theta_{in}=-\tan(\omega t)$ (s22)

It implies that the transverse *E*-fields at various locations of a cross section have the identical polarization direction since the polarization direction is independent on the *ϕ*. However, the polarization direction rotates with the increasing of the time *t*, corresponding to circularly polarized modes in traditional fibers. As can be seen in Eq. (s21), the polarization direction not only rotates with the increasing *t*, but also changes with the increasing *ϕ* since. The EH_+1_ SPPs is thus a vector mode, so-called quasi-elliptically polarized (QEP) mode.

**S 4.1.2 Outside the nanowire**

The azimuthal *E*-fields inside and outside the nanowire have the same polarized direction, however the polarized directions of the radial *E*-fields are opposite. As a consequence, the phase of (azimuthal *E*-fields outside nanowire) is π/2 smaller than the$E_{r}^{out}$ (radial *E*-field outside nanowire) so the horizontal and perpendicular *E*-fields is expressed as

(s23)

and

(s24)

the polarization angle *θ_out_* satisfies

(s25)

**S 4.2 EH-_1_ SPPs**

**S 4.2.1 Inside the nanowire**

Analogous to EH_+1_ SPPs, the horizontal and perpendicular *E*-fields of the EH-_1_ SPPs are written as

(s26)

and

(s27)

Then the polarization angle *θ_in_* is shown as

(s28)

**S 4.2.2 Outside the nanowire**

The polarized directions of azimuthal *E*-fields inside and outside the nanowire are the same, but those of the radial *E*-fields are different. Therefore, the phase of (azimuthal *E*-fields outside nanowire) is π/2 larger than that of $E_{r}^{out}$ (radial *E*-field outside nanowire), so we express the horizontal and perpendicular *E*-fields as

(s29)

and

(s30)

Hence, the polarization angle *θ_out_* is shown by

(s31)

Herein, the EH_-1_ SPPs is also a QEP mode.

**S 4.3 Coherent overlap of the EH_+1_ and EH-_1_ SPPs**

**S 4.3.1 Inside the nanowire**

If the EH_+1_ and EH_-1_ modes, exhibiting both the same intensity and phase in *ϕ* = 0 direction, coherently superpose, the horizontal and perpendicular *E*-fields can be expressed as

(s32)

and

(s33)

So the polarization angle *θ_in_* is expressed as

(s34)

At , Eq.(s34) can be further simplified into

$\tan\theta_{in}=0$ (s35)

Therefore, the polarization directions are not associated with neither *ϕ* nor *t*, showing that the mode is linearly polarized that corresponds to the linearly polarized mode in traditional fibers. In the GCG nanowire, according to Eq. (s34), although the polarization directions of the EH SPPs are independent on *t*, it depends on *ϕ*. This is a vector mode, whereas not linearly polarized, so-called quasi-linearly polarized (QLP) mode.

**S 4.3.2 Outside the nanowire**

The horizontal and perpendicular *E*-fields for the coherent superposition of EH_+1_ and EH_-1_ modes can be shown as

(s36)

and

(s37)

Therefore, the polarization angle *θ_out_* is shown as

(s38)

**S 5. The propagation characteristics of EH_±1_ and TM SPPs in the graphene coated amorphous Ge_2_Sb_2_Te_5_ nanowire at *E_F_* =0.46 eV and 0.6 eV**

In cylindrical coordinates, the longitudinal electric (*E-*) and magnetic (*H-*) fields take the forms and accordingly, where *j* = “in” or “out” represents the region inside the GST rod and outside the graphene shell, *m* the azimuthal quantum number *m* = 0, ±1, ±2,…, $A_{E}^{j}$and $A_{H}^{j}$ mode coefficients, *ω* the angular frequency, $\beta=\beta_{1}+\beta_{2}i$ the complex propagation constant, $h_{j}^{2}=\beta^{2}-\mu_{j}\varepsilon_{j}k_{0}^{2}$ , *k_0_* = 2π /λ, the wave number in vacuum, and *μ_in_* = *μ_out_* = 1 (namely, *μ_a-GST_* = *μ_gel_* = 1) for non-magnetic dielectrics.$Z_{m}^{j}(h_{j}r)$ represents the modified Bessel function$I_{m}(h_{in}r)$ *j* = “in” or $K_{m}(h_{out}r)$ at *j* = “out”. The transverse field components$E_{r}^{j}$,, $H_{r}^{j}$ and are retrieved from $E_{z}^{j}$ , $H_{z}^{j}$, and the relationships between the longitudinal and transverse parts of EM fields [67]. Using the boundary conditions $E_{z}^{in}$ =$E_{z}^{out}$, = , $H_{z}^{out}$- $H_{z}^{in}$ = −*σ_g_* and - = *σ_g_*$E_{z}^{in}$ at *r* = *a*, one can derive the dispersion equation of guide modes [38],

$\begin{matrix} \frac{\frac{1}{h_{in}h_{out}}\frac{I'_{m}(h_{in}a)}{I_{m}(h_{in}a)}\frac{K'_{m}(h_{out}a)}{K_{m}(h_{out}a)}-i\frac{\omega\varepsilon_{0}}{\sigma_{\text{g}}h_{in}k_{0}^{2}}\frac{I'_{m}(h_{in}a)}{I_{m}(h_{in}a)}+i\frac{\omega\varepsilon_{0}}{\sigma_{\text{g}}k_{0}^{2}h_{out}}\frac{K'_{m}(h_{out}a)}{K_{m}(h_{out}a)}}{\frac{1}{h_{out}}\frac{K'_{m}(h_{out}a)}{K_{m}(h_{out}a)}+i\frac{\omega\varepsilon_{0}(\varepsilon_{gel}-\varepsilon_{a-GST})}{\sigma_{\text{g}}h_{out}^{2}}} \\ \times\frac{i\frac{\beta^{2}m^{2}\sigma_{\text{g}}}{\omega\varepsilon_{0}h_{in}^{2}h_{out}^{2}a^{2}}+\frac{\varepsilon_{a-GST}}{h_{in}}\frac{I'_{m}(h_{in}a)}{I_{m}(h_{in}a)}-\frac{\varepsilon_{gel}}{h_{out}}\frac{K'_{m}(h_{out}a)}{K_{m}(h_{out}a)}+i\frac{\sigma_{\text{g}}}{\omega\varepsilon_{0}}}{i\frac{\sigma_{\text{g}}}{\omega\varepsilon_{0}h_{in}}\frac{I'_{m}(h_{in}a)}{I_{m}(h_{in}a)}-\frac{\varepsilon_{gel}-\varepsilon_{a-GST}}{h_{in}^{2}}}=(\frac{m\beta}{h_{in}h_{out}a})^{2} \end{matrix}$ (s39)

When *m* = 0, Eq. (39) can be reduced to

$i\omega\varepsilon_{0}[\frac{\varepsilon_{a-GST}}{h_{in}}\frac{I_{1}(h_{in}a)}{I_{0}(h_{in}a)}+\frac{\varepsilon_{gel}}{h_{out}}\frac{K_{1}(h_{out}a)}{K_{0}(h_{out}a)}]=\sigma_{\text{g}}$ (s40)

$-i\frac{\omega\varepsilon_{0}}{k_{0}^{2}}[h_{in}\frac{I_{0}(h_{in}a)}{I_{1}(h_{in}a)}+h_{out}\frac{K_{0}(h_{out}a)}{K_{1}(h_{out}a)}]=\sigma_{\text{g}}$ (s41)

The dispersion equations of TM and TE SPPs are shown by Eq.(s40) and Eq.(s41), respectively, where the TE SPPs are ignored owing to its weak confinement. Eq. (s42) is a dispersion equation of the EH modes with the lowest order: EH_+1_ and EH_-1_, degenerated by Eq. (s39)

$\begin{matrix} \frac{\frac{1}{4h_{in}h_{out}}\frac{I_{0}(h_{in}a)+I_{2}(h_{in}a)}{I_{1}(h_{in}a)}\frac{K_{0}(h_{out}a)+K_{2}(h_{out}a)}{K_{1}(h_{out}a)}+i\frac{\omega\varepsilon_{0}}{2\sigma_{\text{g}}h_{in}k_{0}^{2}}\frac{I_{0}(h_{in}a)+I_{2}(h_{in}a)}{I_{1}(h_{in}a)}+i\frac{\omega\varepsilon_{0}}{2\sigma_{\text{g}}k_{0}^{2}h_{out}}\frac{K_{0}(h_{out}a)+K_{2}(h_{out}a)}{K_{1}(h_{out}a)}}{\frac{1}{2h_{out}}\frac{K_{0}(h_{out}a)+K_{2}(h_{out}a)}{K_{1}(h_{out}a)}-i\frac{\omega\varepsilon_{0}(\varepsilon_{gel}-\varepsilon_{a-GST})}{\sigma_{\text{g}}h_{out}^{2}}} \\ \times\frac{i\frac{\sigma_{\text{g}}\beta^{2}}{\omega\varepsilon_{0}h_{in}^{2}h_{out}^{2}a^{2}}+i\frac{\sigma_{\text{g}}}{\omega\varepsilon_{0}}+\frac{\varepsilon_{a-GST}}{2h_{in}}\frac{I_{0}(h_{in}a)+I_{2}(h_{in}a)}{I_{1}(h_{in}a)}+\frac{\varepsilon_{gel}}{2h_{out}}\frac{K_{0}(h_{out}a)+K_{2}(h_{out}a)}{K_{1}(h_{out}a)}}{i\frac{\sigma_{\text{g}}}{2\omega\varepsilon_{0}h_{in}}\frac{I_{0}(h_{in}a)+I_{2}(h_{in}a)}{I_{1}(h_{in}a)}-\frac{(\varepsilon_{gel}-\varepsilon_{a-GST})}{h_{in}^{2}}}=(\frac{\beta}{h_{in}h_{out}a})^{2} \end{matrix}$ (s42)

The GST nanowire is 5 nm in radius (*a* = 5 nm) with an amorphous state. The complex propagation constants$\beta=\beta_{1}+\beta_{2}i$ of TM and EH_±1_ SPPs are calculated using Eqs. (s40) and (s42) for *E_F_* = 0.46 eV and 0.6 eV, respectively. Controlling of the *E_F_* of the graphene coating can modulate the profile of the output light. This allows the different functionalities based on either helix or ring output beams to be written into the nanowire without changing its structural geometry. In Fig.S3, we figure the spectra of$\beta_{1}$and$\beta_{2}$.

In the left column of Fig. S3(a), we show that there are modal confinements in both EH_±1_ ($\beta_{1}^{EH_{\pm1}}$) and TM ($\beta_{1}^{TM}$) modes since their$\beta_{1}$ are larger than the wave vector of surrounding ion-gel medium. The $\Delta\beta_{1}=\beta_{1}^{EH_{\pm1}}-\beta_{1}^{TM}$is shown in the right column, where $\Delta\beta_{1}$ is around zero at the mode phase matching (MPM) wavelengths of 4 μm and 7μm with *E_F_*=0.46 eV (red dashed line). It means the phase velocities of EH_±1_ and TM SPP modes are the same. Thereby, the superposition of TM and EH_±1_ modes leads to a stable guiding beam (i.e. a ring beam in the nanowire terminus). However, the *λ_MPM_* blueshifts to 3.5μm at *E_F_* = 0.6 eV hence$\Delta\beta_{1}$is not zero at *λ*=4 μm (pink dashed line). The interaction of EH_±1_ and TM modes is not stable that provides the helix crescent beam. In the left column of Fig. S3(b), we show the EH_±1_ and TM modes can transport a micro-scale distance along the GCG nanowire since their $\beta_{2}$ are around 10^5^ /m. In the right column, the spectra of $\Delta\beta_{2}=\beta_{2}^{EH_{\pm1}}-\beta_{2}^{TM}$are presented, accordingly. As can be observed, the overlap of $\beta_{2}$ between EH_±1_ ($\beta_{2}^{EH_{\pm1}}$) and TM ($\beta_{2}^{TM}$) modes are well maintained from 3μm to 4.5 μm, implying a considerable mode interference.

Fig. S3. Comparisons of propagation characteristics between EH_±1_ and TM SPPs. (a) Left column: the$\beta_{1}$ of EH_±1_ and TM modes. Right column: The differences of the$\beta_{1}$ between the two modes. (b) Left column: the $\beta_{2}$ of EH_±1_ and TM modes. Right column: the differences of the$\beta_{2}$ between the two modes.

**S6. The models built by COMSOL**

Herein, the graphene is treated as atom thick anisotropic layer with a thickness of *T_g_* = 0.5 nm. The expression of the permittivity of the graphene is shown in the primary manuscript. The GST nanowire is 5 nm in radius and has significant change of the complex permittivity (Fig. S1). In the models, we place ports at both the input and output cross sections, where a pair of ports corresponds to one boundary mode. The type of the port is “Numeric”. The other outer surfaces are defined as “scattering boundary condition”. The port is divided by “free triangular”. The meshes for the whole structure are divided using the “swept”. The maximum and minimum element sizes for the “swept” are 3 and 0.3 nm, accordingly. “Boundary Mode Analysis” is employed to calculate two orthotropic QLP EH SPPs and one TM SPPs. The incident light is constructed by the superposition of these modes. The “port phase” is 0 for the TM mode, however in order to create a QEP mode via the coherent superposition of the two QLP EH modes, the “port phase” is 0 for one QLP EH mode and π / 2 for the other. The “port input power” are 1 W for the two QLP EH modes and 4 W for the TM mode so that the maximum intensities of *E*-fields inside the GCG nanowire are the same for the two modes. We can then obtain the best destructive interference along the azimuthal direction, presented by <*S*> at *z* = 0 nm (Fig. S2). Afterwards, the “Optics>Wave Optics>Electromagnetic Waves, Beam Envelopes (ewbe)” corresponding to the “swept” mesh division is performed. Finally, we output and redraw <*S*> at the output cross sections at the different conditions (Fig. 1(b)-(c)).

**S7. The chirality of the emissive light for the amorphous GCG nanowire.**

Fig.S4. The spectra of the degree of circular polarization *C* for the amorphous GCG nanowire.

**S8. The material thermoelectric properties**

In Table S1, we summarize thermoelectric properties for GST, graphene, and ion-gel. The ion-gel is selected as a mixture between SiO_2_ and imidazolium [s1], which possesses an index-matching with SiO_2_.

Table S1. Material thermal properties used in the Heat transfer model

| $\begin{matrix} \text{Special heat} \\ \text{capacity CS} \\ (J/(Kg\cdot K)) \end{matrix}$ $\begin{matrix} \text{Density} \\ \text{ρ(kg/m}^{3}\text{)} \end{matrix}$ $\begin{matrix} \text{Thermal} \\ \text{conductivity} \\ \text{k(W/(m}\cdot\text{k))} \end{matrix}$ $\begin{matrix} \text{Electrical} \\ \text{conductivity} \\ \text{sigma(S/m)} \end{matrix}$ $\begin{matrix} \text{Relative} \\ \text{permittivity} \\ \text{epsilnr(1)} \end{matrix}$ $\begin{matrix} \text{Seebeck} \\ \text{coefficient} \\ \text{S(V/K)} \end{matrix}$ |
| --- |
| **Au** $129^{[s2]}$ $19300^{[s2]}$ $\begin{matrix} \text{ 317(bulk)}^{\text{[s2]}}\text{,} \\ \text{20 Thickness } \\ \text{ 5nm}^{[s2]} \end{matrix}$ $\begin{matrix} \text{ Temperature} \\ \text{dependence}^{\text{[s3]}} \end{matrix}$ $6.9^{[s4]}$ $\begin{matrix} \text{ Temperature} \\ \text{dependence}^{\text{[s5]}} \end{matrix}$ $\text{Ge}_{2}\text{Sb}_{2}\text{Te}_{5}$ $220^{[s6]}$ $6150^{[s7]}$ $\begin{matrix} \text{ Temperature} \\ \text{dependence}^{[s7]} \end{matrix}$ $\begin{matrix} \text{ Temperature} \\ \text{dependence}^{\text{[s8]}} \end{matrix}$ $\begin{matrix} \text{ Temperature} \\ \text{dependence}^{\text{[s9]}} \end{matrix}$ $\begin{matrix} \text{ Temperature} \\ \text{dependence}^{\text{[s10]}} \end{matrix}$ $Graphene$ $700^{[s11]}$ $1060^{[s12]}$ $3000^{\text{[s13]}}$ $1400^{\text{[s14]}}$ $67.8^{\text{[s15]}}$ $0.00003^{\text{[s16]}}$ $Ion-gel$ $4.4277^{[s17]}$ $1299^{[s18]}$ $0.117^{[s19]}$ $0.4^{\text{[s19]}}$ $2.25^{\text{[38]}}$ $0.00094^{\text{[s19]}}$ |

[38] Liang H, Zhang L, Zhang S, et al. Gate-Programmable Electro-Optical Addressing Array of Graphene-Coated Nanowires with Sub-10 nm Resolution. *ACS Photonics*, 2016; **3**: 1847-1853.

[67] Snitzer E. Cylindrical dielectric waveguide modes. *Journal of the Optical Society of America*, 1961; **51**: 491-498.

1. Maréchal M, Laberty-Robert C, Livi S. Hybrid Electrolytes[M]/ Nanomaterials for Sustainable Energy. *American Chemical Society*, 2015; 73-97.
2. Chen G, Hui P. Thermal conductivities of evaporated gold films on silicon and glass. *Applied physics letters*, 1999; **74**: 2942-2944.
3. Haynes W M, Lide D R. Handbook of chemistry and physics. National Institute for Standards and Technology, CRC Press, New York, NY, 2010.
4. Shklyarevskii I N, Pakhomov P L. Separation of Contributions from Free and Coupled Electrons into Real and Imaginary Parts of Dielectric-Constant of Gold. *Optika i Spektroskopiya*, 1973; **34**: 163-166.
5. Matula R A. Electrical resistivity of copper, gold, palladium, and silver. *Journal of Physical and Chemical Reference Data*, 1979; **8**: 1147-1298.
6. Kuwahara M, Suzuki O, Yamakawa Y, et al. Measurement of the thermal conductivity of nanometer scale thin films by thermoreflectance phenomenon. *Microelectronic engineering*, 2007; **84**: 1792-1796.
7. Lyeo H K, Cahill D G, Lee B S, et al. Thermal conductivity of phase-change material Ge_2_Sb_2_Te_5_. *Applied Physics Letters*, 2006; **89**: 151904.
8. Kim Y K, Hwang U, Cho Y J, et al. Change in electrical resistance and thermal stability of nitrogen incorporated Ge 2 Sb 2 Te 5 films. *Applied physics letters*, 2007; **90**: 021908.
9. Kuwahara M, Suzuki O, Taketoshi N, et al. Measurements of temperature dependence of optical and thermal properties of optical disk materials. *Japanese journal of applied physics*, 2006; **45**: 1419.
10. Kurinec S K, Iniewski K. Nanoscale Semiconductor Memories: Technology and Applications. CRC Press, 2013.
11. Pop E, Varshney V, Roy A K. Thermal properties of graphene: Fundamentals and applications. *MRS bulletin*, 2012; **37**: 1273-1281.
12. Rafiee M A, Rafiee J, Wang Z, et al. Enhanced mechanical properties of nanocomposites at low graphene content. *ACS nano*, 2009; **3**: 3884-3890.
13. Balandin A A. Thermal properties of graphene and nanostructured carbon materials. *Nature Materials*, 2011; **10**: 569-581.
14. Marinho B, Ghislandi M, Tkalya E, et al. Electrical conductivity of compacts of graphene, multi-wall carbon nanotubes, carbon black, and graphite powder. *Powder Technology*, 2012; **221**: 351-358.
15. Zhu W, Rukhlenko I D, Si L M, et al. Graphene-enabled tunability of optical fishnet metamaterial. *Applied Physics Letters*, 2013; **102**: 121911.
16. Li X, Yin J, Zhou J, et al. Exceptional high Seebeck coefficient and gas-flow-induced voltage in multilayer graphene. *Applied Physics Letters*, 2012; **100**: 183108.
17. Gardas R L, Freire M G, Carvalho P J, et al. High-pressure densities and derived thermodynamic properties of imidazolium-based ionic liquids. *Journal of Chemical & Engineering Data*, 2007; **52**: 80-88.
18. Harris K R, Kanakubo M, Woolf L A. Temperature and pressure dependence of the viscosity of the ionic liquid 1-butyl-3-methylimidazolium tetrafluoroborate: viscosity and density relationships in ionic liquids. *Journal of Chemical & Engineering Data*, 2007; **52**: 2425-2430.
19. Jia H, Tao X, Wang Y. Flexible and Self‐Healing Thermoelectric Converters Based on Thermosensitive Liquids at Low Temperature Gradient. *Advanced Electronic Materials*, 2016; **2**: 1600136.
